# Measuring the Concentration of Serum Syndecan-1 to Assess Vascular Endothelial Glycocalyx Injury During Hemodialysis

**DOI:** 10.3389/fmed.2021.791309

**Published:** 2021-12-23

**Authors:** Keigo Kusuzawa, Keiko Suzuki, Hideshi Okada, Kodai Suzuki, Chihiro Takada, Soichiro Nagaya, Ryu Yasuda, Haruka Okamoto, Takuma Ishihara, Hiroyuki Tomita, Yuki Kawasaki, Toru Minamiyama, Ayane Nishio, Hirotsugu Fukuda, Takuto Shimada, Yuto Tamaoki, Tomoki Yoshida, Yusuke Nakashima, Naokazu Chiba, Genki Yoshimura, Ryo Kamidani, Tomotaka Miura, Hideaki Oiwa, Fuminori Yamaji, Yosuke Mizuno, Takahito Miyake, Yuichiro Kitagawa, Tetsuya Fukuta, Tomoaki Doi, Akio Suzuki, Takahiro Yoshida, Nobuyuki Tetsuka, Shozo Yoshida, Shinji Ogura

**Affiliations:** ^1^Department of Emergency and Disaster Medicine, Gifu University Graduate School of Medicine, Gifu, Japan; ^2^Department of Infection Control, Gifu University Graduate School of Medicine, Gifu, Japan; ^3^Innovative and Clinical Research Promotion Center, Gifu University Hospital, Gifu, Japan; ^4^Department of Tumor Pathology, Gifu University Graduate School of Medicine, Gifu, Japan; ^5^Abuse Prevention Center, Gifu University Graduate School of Medicine, Gifu, Japan; ^6^Department of Pharmacy, Gifu University Hospital, Gifu, Japan

**Keywords:** hemodialysis, glycocalyx, syndecan-1, body fluid removal, nafamostat mesylate

## Abstract

Glycocalyx is present on the surface of healthy endothelium, and the concentration of serum syndecan-1 can serve as an injury marker. This study aimed to assess endothelial injury using serum syndecan-1 as a marker of endothelial glycocalyx injury in patients who underwent hemodialysis. In this single-center, retrospective, observational study, 145 patients who underwent hemodialysis at the Gifu University Hospital between March 2017 and December 2019 were enrolled. The median dialysis period and time were 63 months and 3.7 h, respectively. The serum syndecan-1 concentration significantly increased from 124.6 ± 107.8 ng/ml before hemodialysis to 229.0 ± 138.1 ng/ml after hemodialysis (*P* < 0.001). Treatment with anticoagulant nafamostat mesylate inhibited hemodialysis-induced increase in the levels of serum syndecan-1 in comparison to unfractionated heparin. Dialysis time and the change in the syndecan-1 concentration were positively correlated. Conversely, the amount of body fluid removed and the changes in the syndecan-1 concentration were not significantly correlated. The reduction in the amount of body fluid removed and dialysis time inhibited the change in the syndecan-1 levels before and after hemodialysis. In conclusion, quantitative assessment of the endothelial glycocalyx injury during hemodialysis can be performed by measuring the serum syndecan-1 concentration, which may aid in the selection of appropriate anticoagulants, reduction of hemodialysis time, and the amount of body fluid removed.

## Introduction

Malnutrition, inflammation, and atherosclerosis are strongly associated with each other in chronic kidney disease (1), and both a malnourished state and atherosclerosis can be caused by inflammation. Moreover, chronic microinflammation is observed in patients who undergo hemodialysis (2). Several factors, such as uremia, activation of free radicals and adhesion molecules, and hemodialysis membrane, can lead to microinflammation in patients who undergo hemodialysis (3–5). Uremic substances and the hemodialysis membrane promote the production of free radicals and cytokines by stimulating neutrophils, and the resulting inflammation further causes endothelial injury. However, there is no method to quantitatively assess endothelial cell injury.

The vascular endothelium is composed of a thin monolayer of endothelial cells, and this lines the entire circulatory system, particularly the parts that are exposed to the circulating blood. The healthy endothelium is covered by a glycoprotein called glycocalyx (6–10), which plays pivotal roles in vascular homeostasis (11, 12). The endothelial glycocalyx is degraded by several factors, such as sepsis, major surgery, trauma, ischemia/reperfusion, and prolonged hyperglycemia. Persistent and diffuse alterations in the glycocalyx are associated with widespread endothelial dysfunction, changed permeability, and impaired oxygen and nutrient delivery to the cells (11, 13, 14). Several studies have revealed the relationship between endothelial glycocalyx injury and serious diseases, such as cardiovascular disease, acute kidney injury, and chronic kidney disease (15, 16). Moreover, the structure of the endothelial glycocalyx is degraded in chronic diseases, such as aging (17), diabetes (18), and hypertriglyceridemia (19).

The glycocalyx comprises cell-bound proteoglycans, glycosaminoglycan side chains, and sialoproteins (13, 20). The proteoglycans consist of a core protein, such as a member of the syndecan protein family, to which glycosaminoglycan molecules are linked (21). Syndecan-1 is the core protein in heparan sulfate proteoglycan that is observed in the glycocalyx. It is released from the endothelium when the glycocalyx is injured, causing an increase in its concentration in circulation (22). Moreover, serum syndecan-1 has been used as an endothelial injury marker in recent clinical studies in critically ill patients (23–26).

Therefore, the present study aimed to assess endothelial injury using the serum syndecan-1 level as a marker of endothelial glycocalyx injury in patients who underwent hemodialysis. Additionally, we examined the medication type and factors that influence the concentration of syndecan-1.

## Methods

### Patients

**Figure 1:**
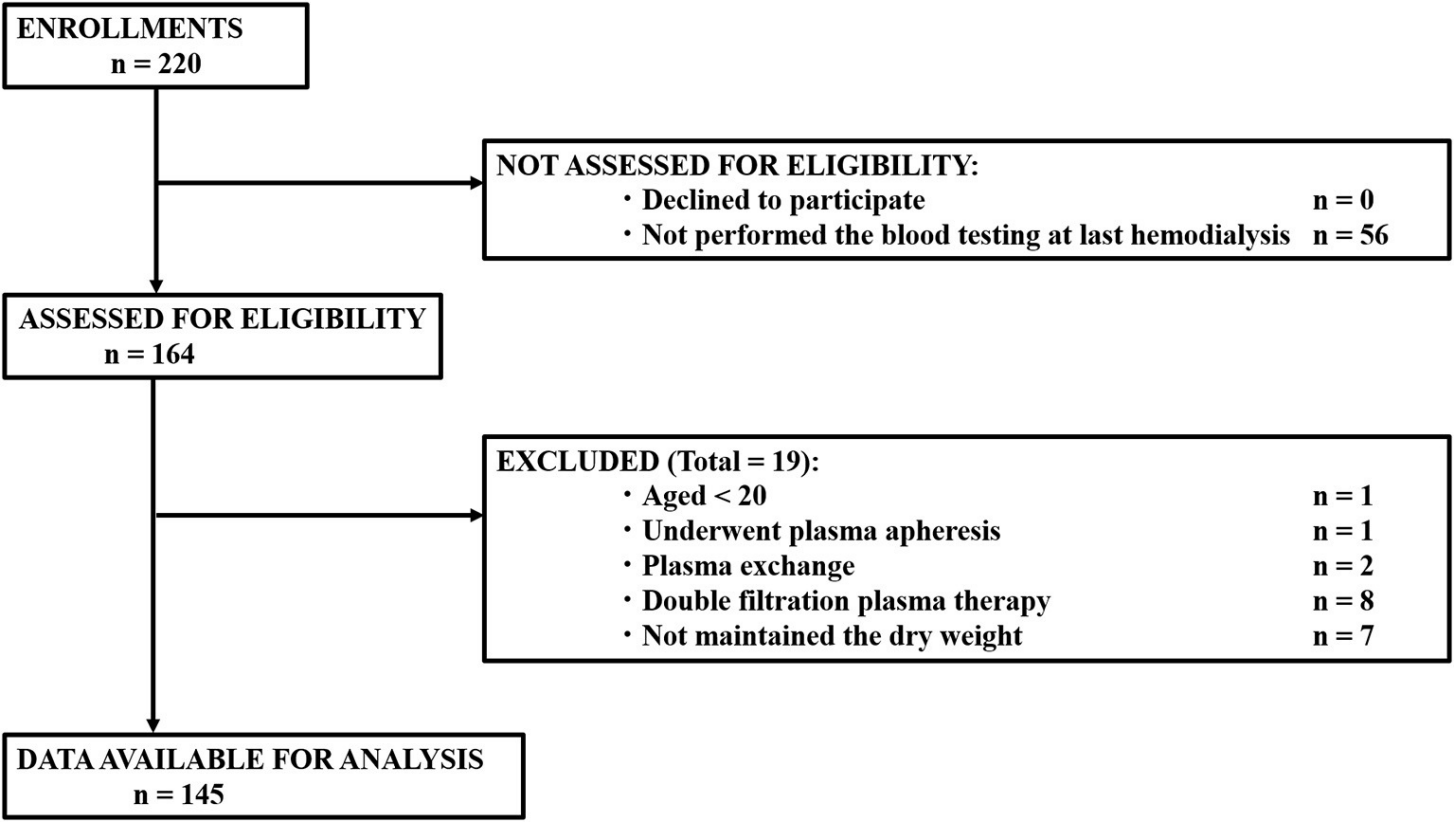
Flow diagram for the selection of study participants for data analysis.

This was a single-center, retrospective, observational study conducted at the Gifu University Hospital, affiliated to Gifu University, Gifu, Japan. Patients, who underwent hemodialysis at the Gifu University Hospital between March 2017 and December 2019 and whose dry weight remained unchanged in the last three examinations, were enrolled. Patients aged <20 years, who underwent plasma apheresis, plasma exchange, and double filtration plasma therapy, and had not maintained their dry weight were excluded. Finally, data from 145 patients were obtained and analyzed (Figure 1).

### Ethics Approval and Consent to Participate

The study conformed to the principles outlined in the Declaration of Helsinki (43). Ethics approval was obtained from the Medical Ethics Committee of the Gifu University Graduate School of Medicine, Gifu, Japan (approval no.: 2021-A005). The need for informed consent from participants was waived by the medical ethics committee because of the retrospective nature of the study. Before initiation, the study was registered in the UMIN Clinical Trials Registry (registry number: UMIN000051415).

### Data Collection and Study Design

Blood was routinely sampled before and after hemodialysis from eligible patients at the time of the last hemodialysis before being discharged from Gifu University Hospital, and data from these blood samples were used in the present study. All laboratory data (except serum syndecan-1), dry weight, and other patient demographics were extracted from the hospital's electronic medical records. The concentration of serum syndecan-1 was measured using an enzyme-linked immunosorbent assay (950.640.192; Diaclone, Besancon, Cedex, France). The data were retrospectively analyzed. As an index of the efficiency of dialysis, Kt/V was calculated as described previously (44).

### Statistical Analysis

The sample size in this study was calculated to obtain sufficient amount of data and conditions to avoid overfitting in multivariable regression analysis. It was necessary to collect at least 90 patients to estimate the parameters of the six covariates, including the interaction term in the multivariable regression model (45). Patients' baseline characteristics are presented as medians and interquartile ranges for continuous variables, and as frequencies and proportions for categorical variables. For the primary analysis, a mixed effect model was used to assess the change in the syndecan-1 levels with hemodialysis. The difference in the syndecan-1 levels before and after hemodialysis in the anticoagulant subgroup was confirmed using paired *t*-test. A multivariable linear regression analysis was performed to compare the change in the syndecan-1 levels before/after hemodialysis and treatment with anticoagulants. The covariates in the regression model were age, sex, dry weight, and dialysis period (46). These variables were selected *a priori* based on previous studies. In another model, dry weight and dialysis period were simultaneously incorporated into the linear regression model to evaluate the effect of factors during dialysis on the levels of syndecan-1. An interaction term was included in the model to confirm the effect of modification of dry weight and dialysis period on changes in the levels of syndecan-1. If the interaction term was statistically significant, the effect of the dialysis period (or dry weight) on syndecan-1 was determined to be modified by dry weight (or the dialysis period). There were no missing values in the data used in the analyses. A two-sided *P*-value < 0.05 was considered to be statistically significant. The study was exploratory and there were concerns concerning the low statistical power; therefore, the interaction was evaluated with a two-sided *P* < 0.1. R version 4.1.0 was used for statistical analyses (R Foundation for Statistical Computing, Vienna, Austria).

## Results

### Characteristics of Patients

We finally enrolled 145 patients with a median age of 66 years (Figure 1; Table 1). The median dialysis period and time were 63 months and 3 h and 45 min, respectively. The most common primary illness was diabetic nephropathy, which was observed in 37 patients (25.5%).

**Table 1:** Patient demographics.

**Characteristics**	**Median (range) or number**
Number of cases, *n*	145
Age (years), mean (IQR)	68 (60–77)
Sex (female/male), *n* (%)	44 (30.3)/101 (69.7)
Dialysis period (months), median (IQR)	20.0 (1.0–87.0)
Dialysis time (h), median (IQR)	4.0 (3.0–4.0)
Systolic blood pressure before dialysis (mmHg), median (IQR)	136.0 (121.0–157.0)
Diastolic blood pressure before dialysis (mmHg), median (IQR)	69.0 (59.0–79.0)
Primary illness, *n* (%)	
Chronic glomerulonephritis	29 (20.0)
Rapidly progressive glomerulonephritis	4 (2.8)
Polycystic kidney disease	8 (5.5)
Nephrosclerosis	8 (5.5)
Diabetic nephropathy	37 (25.5)
Nephritis with autoimmune disease	6 (4.1)
Renal/urological tumor	5 (3.4)
Obstructive urinary tract/urination disorders	1 (0.7)
Paraproteinemia (myeloma)	1 (0.7)
Acute kidney injury	10 (6.9)
Congenital anomalies of the kidney and urinary tract	1 (0.7)
Unknown	33 (22.8)
Others	2 (1.4)
Hemodialysis type, *n* (%)	
HD	133 (91.7)
HDF	12 (8.3)
Vascular access type, *n* (%)	
Arteriovenous fistula	107 (73.8)
Arteriovenous graft	8 (5.5)
Temporary vascular catheter	23 (15.9)
Permanent vascular catheter	7 (4.8)
Dialysis efficiency	
Kt/V	1.20 (0.06–1.86)
Medication, *n* (%)	
Unfractionated heparin	101 (69.7)
Low-molecular-weight heparin	24 (16.6)
Nafamostat mesylate	20 (13.8)
Dialysis membrane, *n* (%)	
Polyethersulfone	110 (75.9)
Polysulfone	34 (23.4)
Asymmetric triacetate	1 (0.7)

*HD, hemodialysis; HDF, hemodiafiltration; IQR, interquartile range*.

The number of patients who underwent hemodialysis and hemodiafiltration was 133 (91.7%) and 12 (8.3%), respectively. Anticoagulation agents, such as unfractionated heparin, low-molecular-weight heparin, and nafamostat mesylate, were administered to 101, 24, and 20 patients, respectively. The median Kt/V-value, an index of dialysis efficiency, was 1.20.

### Concentration of Serum Syndecan-1 and Hemodialysis

The concentrations of serum syndecan-1 before and after hemodialysis were 124.6 ± 107.8 and 229.0 ± 138.1 ng/ml, respectively; this indicated that the serum syndecan-1 concentration significantly increased (*P* < 0.001) after hemodialysis (Table 2).

**Table 2:** Serum SDC-1 concentration before and after hemodialysis.

**Status**	**SDC-1 concentration (ng/ml)**	***P*-value**
Before HD	124.6 ± 107.8	<0.001
After HD	229.0 ± 138.1	

*SDC-1, syndecan-1; HD, hemodialysis. P-values were obtained from a mixed-effects model*.

The concentration of serum syndecan-1 significantly increased after hemodialysis in patients who received unfractionated heparin and low-molecular-weight heparin; however, the concentration of syndecan-1 was not significantly different before and after hemodialysis in those who received nafamostat mesylate (Table 3).

**Table 3:** Serum SDC-1 concentration and anticoagulants.

**Anticoagulants**	**Before HD (ng/ml)**	**After HD (ng/ml)**	***P*-value**
Unfractionated heparin	112.0 ± 79.8	235.4 ± 126.8^*^	<0.001
Low-molecular-weight heparin	144.1 ± 135.8	248.5 ± 174.1^*^	<0.001
Nafamostat mesylate	164.2 ± 171.1	173.3 ± 141.3	0.459

*SDC-1, syndecan-1; HD, hemodialysis*.

* *Statistically significant (P < 0.05)*.

Additionally, according to the multivariable regression analysis after adjusting for age, sex, dry weight, and dialysis period, the treatment with nafamostat mesylate inhibited the increase in the concentration of serum syndecan-1 during hemodialysis compared to treatment with unfractionated heparin and low-molecular-weight heparin (Table 4).

**Table 4:** Results of multivariable regression analysis between anticoagulants.

**Anticoagulants**	Coefficient^*^	**95% LCL**	**95% UCL**	***P*-value**
Low-molecular-weight heparin vs. unfractionated heparin	−18.07	−56.776	20.637	0.358
Nafamostat mesylate vs. unfractionated heparin	−116.473	−158.442	−74.504	<0.001
Nafamostat mesylate vs. low-molecular-weight heparin	−98.403	−150.482	−45.324	<0.001

*LCL, lower confidence limit; UCL, upper confidence limit*.

* *Coefficients from the multivariable linear regression model adjusted for age, sex, dry weight, and dialysis period, shown as differences in the serum syndecan-1 concentration for low-molecular-weight heparin vs. unfractionated heparin, nafamostat mesylate vs. unfractionated heparin, and nafamostat mesylate vs. low-molecular-weight heparin, respectively*.

Interestingly, there was no strong relationship between the syndecan-1 levels and blood pressure (Supplementary Table 1), vascular access (Supplementary Table 2), cardiovascular disease (Supplementary Table 3), and primary disease (Supplementary Table 4) before and after dialysis.

### Association of Concentration of Serum Syndecan-1 With Dialysis Time and Body Fluid Removal

The relationship between the concentration variability of syndecan-1 and the dialysis condition, including the dialysis time and the amount of body fluid removed, was confirmed. The amount of body fluid removal was corrected by the dry weight. The dialysis time and change in concentration of syndecan-1 showed a positive correlation (*P* = 0.033), but there was no significant association (*P* = 0.111) between the amount of body fluid removed and the syndecan-1 concentration changes (Table 5).

**Table 5:** Relationship between syndecan-1 concentration variability and dialysis conditions.

**Factors**	Coefficient^*^	**95% LCL**	**95% UCL**	***P*-value**
Body fluid removed/dry weight (per 0.01 L/kg increase)	9.107	0.144	18.07	0.111
Dialysis time (per 1 min increase)	23.349	−8.836	55.533	0.033

*LCL, lower confidence limit; UCL, upper confidence limit*.

* *Coefficients from the multivariable linear regression model adjusted for age, sex, and dialysis period, shown as increment in the serum syndecan-1 concentration for a unit change in factors*.

**Figure 2:**
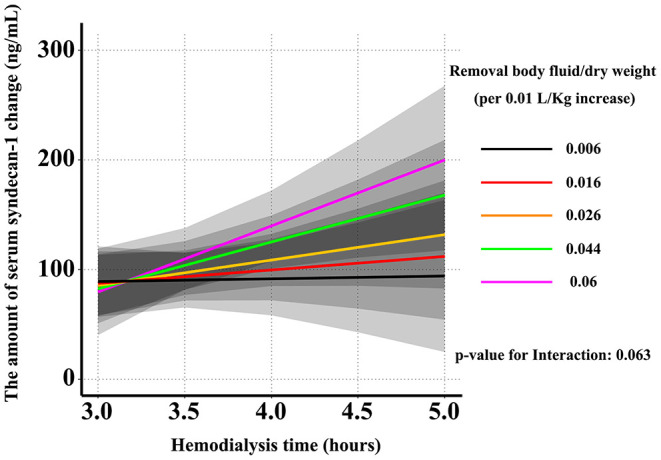
Effect of dialysis time and the amount of body fluid removed on the change in the serum syndecan-1 concentration. The change in the concentration of syndecan-1 before and after hemodialysis increased with respect to the enhanced body fluid removal and prolonged dialysis time. However, the change in the concentration of syndecan-1 before and after hemodialysis decreased with respect to the decreased amount of body fluid removal and the shortened dialysis time.

Next, we examined the modifying effect of the amount of body fluid removed on the association between the change in the concentration of syndecan-1 and dialysis time. The change in the syndecan-1 concentration before and after hemodialysis increased with respect to enhanced removal of body fluids and prolonged dialysis time (*P* for interaction = 0.063, Figure 2). However, the change in the syndecan-1 concentration before and after hemodialysis decreased with respect to a decrease in the amount of body fluid removed and shortened dialysis time.

## Discussion

The present study revealed the following: (a) the endothelial glycocalyx may be injured during hemodialysis; (b) endothelial glycocalyx injury may be attenuated by administering nafamostat mesylate as an anticoagulant; and (c) endothelial glycocalyx injury may be aggravated by an increase in the amount of body fluid removed and prolonged dialysis time.

A previous study reported that the serum syndecan-1 concentration was approximately 20 ng/ml in healthy individuals (19); in contrast, in this study, it was found to be 124.6 ± 107.8 ng/ml in patients who underwent hemodialysis. This result confirmed that the endothelial glycocalyx sustained injuries in patients who underwent hemodialysis, consistent with the findings of a previous report (27). This indicates that endothelial glycocalyx injury may be aggravated by an increasing fluid volume.

Moreover, the serum syndecan-1 concentration increased after hemodialysis compared to the corresponding value before hemodialysis. This finding suggested that the endothelial glycocalyx was injured during hemodialysis, probably because of the production of free radicals and cytokines during this procedure.

During hemodialysis, unfractionated heparin, low-molecular-weight heparin, and nafamostat mesylate were used as anticoagulating agents. Low-molecular-weight heparin and nafamostat were administered to patients that had any disease that was associated with bleeding tendencies. Our results suggested that an increase in the syndecan-1 concentration was attenuated in patients who received nafamostat mesylate.

Nafamostat mesylate, a synthetic serine protease inhibitor, is a short-acting anticoagulant (28), and is also used during hemodialysis to prevent proteolysis of fibrinogen into fibrin (29). It is a slow, tight-binding substrate that traps the target protein in the acyl-enzyme intermediate form and inhibits enzyme activity (30, 31). It was previously reported that nafamostat mesylate can inhibit the kallikrein-kinin system, which promoted vascular permeability via the produced bradykinin (32–34). In addition, nafamostat has been recently identified as a potential therapy against the coronavirus disease (35). Infection with severe acute respiratory syndrome coronavirus 2 induces endotheliitis due to viral involvement and inflammatory response of the host and, thus, it is associated with endothelial glycocalyx injury (21). Therefore, nafamostat mesylate may have a beneficial effect on the endothelial glycocalyx, although this is supported only by circumstantial evidence.

Extension of dialysis time is a strategy to improve prognosis (36); however, it remains controversial (36, 37). The present study identified that changes in the serum syndecan-1 levels are small in patients who have prolonged dialysis time and slow removal of body fluid. Therefore, these two strategies could prevent endothelial glycocalyx injury. Several reports have also revealed that prolonged hemodialysis was associated with improved blood pressure and fluid management (38–40). Additionally, rapid removal of body fluid is associated with a greater risk of mortality and cardiovascular events (41). Moreover, hypotension during hemodialysis is also associated with higher mortality (42).

These mechanisms may explain how lower ultrafiltration rates with prolonged hemodialysis and slow removal of body fluids may ameliorate endothelial vascular permeability via attenuation of endothelial glycocalyx injury. Therefore, we propose that slow removal of body fluids with prolonged hemodialysis can reduce hypotension during hemodialysis.

This study had some limitations. First, the hemodialysis time in most patients was <4 h. Therefore, an accurate examination of prolonged hemodialysis could not be performed. Second, less types of dialyzer were used in the present study. Third, in this study, other biomarkers of glycocalyx injury, such as the serum hyaluronan and hyaluronidase levels, were not measured. Although further studies are required, measuring the concentration of serum syndecan-1 may help assessing endothelial injury under low blood flow (e.g., chronic hemodiafiltration) and membrane compatibility by using a different type of dialyzer. Moreover, the serum syndecan-1 level is proposed to be a useful biomarker for daily monitoring of organ dysfunction, and may be an important risk factor for mortality in critically ill patients (25).

In conclusion, the study presented a method for the quantitative assessment of endothelial glycocalyx injury by measuring the concentration of serum syndecan-1 during hemodialysis. Although hemodialysis causes endothelial glycocalyx injury, it may be mitigated by maintenance of hemodialysis duration and by modulation of the amount of body fluid removed via the quantitative assessment of the serum syndecan-1 level.

## Data Availability Statement

The raw data supporting the conclusions of this article will be made available by the authors, without undue reservation.

## Ethics Statement

Ethics approval was obtained from the Medical Ethics Committee of the Gifu University Graduate School of Medicine, Gifu, Japan (record no.: 2021-A005). Written informed consent for participation was not required for this study in accordance with the national legislation and the institutional requirements.

## Author Contributions

KK and HOkad wrote the manuscript. KK, KoS, SN, HOkam, GY, RK, and TMiu collected the blood samples. FY, HOi, CT, YKa, TMin, AN, HF, TS, YT, ToY, YN, and NC measured the syndecan-1 concentration using ELISA. KeS and RY created a database. TI performed a statistical analysis. YM, TMiy, YKi, TF, TD, and TaY treated the patients. HT, SY, and SO supervised the study. HOkad and AS revised and edited the manuscript. All authors contributed to the article and approved the submitted version.

## Funding

This work was supported by the Ministry of Education, Science and Culture of Japan grants-in-aid for scientific research (grant numbers 19H03756; HOkad, 19K18347; TF, 19K09410; TD, and 18K16511; SY).

## Conflict of Interest

The authors declare that the research was conducted in the absence of any commercial or financial relationships that could be construed as a potential conflict of interest.

## Publisher's Note

All claims expressed in this article are solely those of the authors and do not necessarily represent those of their affiliated organizations, or those of the publisher, the editors and the reviewers. Any product that may be evaluated in this article, or claim that may be made by its manufacturer, is not guaranteed or endorsed by the publisher.
