# Supplementary material for: Measuring the Concentration of Serum Syndecan-1 to Assess Vascular Endothelial Glycocalyx Injury During Hemodialysis
Source: Front Med (Lausanne). 2021 Dec 23;8:791309. doi: 10.3389/fmed.2021.791309 (PMC8733596; doi:10.3389/fmed.2021.791309)
Supplement: Supplementary file 1 [file Data_Sheet_1.docx]

Supplementary Material

**Supplementary Table 1. The relationship between syndecan-1 levels and blood pressure**

|  | Rho | Lower CI | Upper CI | *P*-value |
| --- | --- | --- | --- | --- |
| Pre: systolic BP vs syndecan-1 concentration | 0.02 | -0.14 | 0.19 | 0.78 |
| Pre: diastolic BP vs syndecan-1 concentration | 0.01 | -0.15 | 0.17 | 0.89 |
| Post: systolic BP vs syndecan-1 concentration | -0.06 | -0.22 | 0.1 | 0.446 |
| Post: diastolic BP vs syndecan-1 concentration | -0.09 | -0.25 | 0.08 | 0.29 |

*CI* confidence interval

**Supplementary Table 2. Serum syndecan-1 concentration by vascular access**

| Characteristic | AV fistula, n=107^1^ | AV graft, n=8^1^ | P cath n=107^1^ | T cath, n=23^1^ | *P*-value^2^ |
| --- | --- | --- | --- | --- | --- |
| **Pre syndecan-1 levels** | 95.7 (62.3–31.8) | 91.0 (54.9–312.5) | 95.7 (62.3–131.8) | 88.6 (62.0–111.5) | 0.264 |
| **Post syndecan-1 levels** | 194.2 (135.0–265.4) | 183.7 (121.7–385.0) | 194.2 (135.0–265.4) | 193.7 (153.8–259.8) | 0.806 |
| ^1^Median (IQR); ^2^Kruskal-Wallis rank sum test | | | | | |
| *AV* arteriovenous, *AV* arteriovenous, *P cath* permanent catheter, *T cath* temporary catheter | | | | | |

**Supplementary Table 3. The relationship between the syndecan-1 levels and cardiovascular disease**

| Characteristic | Cardiovascular disease (-), n=79^1^ | Cardiovascular disease (+), n=66^1^ | *P*-value^2^ |
| --- | --- | --- | --- |
| Pre syndecan-1 levels | 85.7 (60.5–120.8) | 106.2 (66.1–151.0) | 0.117 |
| Post syndecan-1 levels | 193.4 (129.9–300.1) | 199.3 (147.9–247.9) | 0.886 |
| ^1^Median (IQR) | | | |
| ^2^Kruskal-Wallis rank sum test | | | |
| Cardiovascular disease included ischemic heart disease, valvular disease, myocarditis, pericarditis, aortic dissection, aortic aneurysm and arteriosclerosis obliterans.  *IQR* interquartile range | | | |

**Supplementary Table 4. Serum syndecan-1 concentration by primary illness**

| Characteristic | Chronic GN, n=92^1^ | DM, n=37^1^ | AKI, n=13^1^ | Other, n=3^1^ | *P*-value^2^ | |
| --- | --- | --- | --- | --- | --- | --- |
| **Pre syndecan-1 levels** | 92.4 (57.6–136.9) | 99.2 (62.2–129.5) | 90.3 (84.2–118.2) | 131.1 (102.1–142.0) | 0.765 | |
| **Post syndecan-1 levels** | 192.7 (130.0–286.5) | 209.0 (137.6–234.6) | 193.7 (170.2–220.0) | 180.1 (169.9–229.0) | 0.949 | |
| ^1^Median (IQR) | | | | | |  |
| ^2^Kruskal-Wallis rank sum test | | | | | |  |

*CGN* glomerulonephritis, *DM* diabetes mellitus, *AKI* acute kidney injury
